# Functional Neuroimaging in Psychiatry—Aiding in Diagnosis and Guiding Treatment. What the American Psychiatric Association Does Not Know

**DOI:** 10.3389/fpsyt.2020.00276

**Published:** 2020-04-15

**Authors:** Theodore A. Henderson, Muriel J. van Lierop, Mary McLean, John Michael Uszler, John F. Thornton, Yin-Hui Siow, Dan G. Pavel, Joe Cardaci, Phil Cohen

**Affiliations:** ^1^ The Synaptic Space, Inc., Denver, CO, United States; ^2^ Neuro-Luminance, Inc., Denver, CO, United States; ^3^ Dr. Theodore Henderson, Inc., Denver, CO, United States; ^4^ International Society of Applied Neuroimaging, Denver, CO, United States; ^5^ Private Practice, Toronto, ON, Canada; ^6^ Private Practice, Toronto, ON, Canada; ^7^ Nuclear Medicine, Providence St. John's Health Center, Santa Monica, CA, United States; ^8^ Molecular and Medical Pharmacology, University of California, Los Angeles, Los Angeles, CA, United States; ^9^ Rossiter-Thornton Associates, Toronto, ON, Canada; ^10^ Nuclear Medicine, Southlake Regional Health Centre, Newmarket, ON, Canada; ^11^ PathFinder Brain SPECT, Deerfield, IL, United States; ^12^ Fremantle-School of Medicine, University of Notre Dame, Fremantle, WA, Australia; ^13^ Diagnostic Nuclear Medicine, Hollywood Private Hospital, Nedlands, WA, Australia; ^14^ Consultant Physician, Perth, WA, Australia; ^15^ Nuclear Medicine, Lions Gate Hospital, Vancouver, BC, Canada; ^16^ Radiology, University of British Columbia, Vancouver, BC, Canada

**Keywords:** SPECT, single photon emission computarized tomography, positron emision tomography (PET), depression, inflammation, herpes, dementia, ADHD

## Abstract

While early efforts in psychiatry were focused on uncovering the neurobiological basis of psychiatric symptoms, they made little progress due to limited ability to observe the living brain. Today, we know a great deal about the workings of the brain; yet, none of this neurobiological awareness has translated into the *practice* of psychiatry. The categorical system which dominates psychiatric diagnosis and thinking fails to match up to the real world of genetics, sophisticated psychological testing, and neuroimaging. Nevertheless, the American Psychiatric Association (APA) recently published a position paper stating that neuroimaging provided no benefit to the diagnosis and treatment of psychiatric disorders. Using the diagnosis of depression as a model, we illustrate how setting aside the unrealistic expectation of a pathognomonic “fingerprint” for categorical diagnoses, we can avoid missing the biological and, therefore, treatable contributors to psychopathology which can and are visualized using functional neuroimaging. Infection, toxicity, inflammation, gut-brain dysregulation, and traumatic brain injury can all induce psychiatric manifestations which masquerade as depression and other psychiatric disorders. We review these and provide illustrative clinical examples. We further describe situations for which single photon emission computed tomography (SPECT) and positron emission tomography (PET) functional neuroimaging already meet or exceed the criteria set forth by the APA to define a neuroimaging biomarker, including the differential diagnosis of Alzheimer's disease and other dementias, the differential diagnosis of ADHD, and the evaluation of traumatic brain injury. The limitations, both real and perceived, of SPECT and PET functional neuroimaging in the field of psychiatry are also elaborated. An important overarching concept for diagnostic imaging in all its forms, including functional neuroimaging, is that imaging allows a clinician to eliminate possibilities, narrow the differential diagnosis, and tailor the treatment plan. This progression is central to any medical diagnostic process.

## Introduction

The medical application of X-rays was discovered by Wilhelm Conrad Roentgen in 1895 (1, 2). Within a decade, x-rays were being used in medicine, increasing dramatically during World War I, and became standard of care within two decades. Gamma radiation was discovered by Paul Villard in 1900 (1, 3). Almost 70 years later, David Kuhl and Roy Edwards harnessed gamma radiation in the first gamma emission tomographs in 1964 (4). Building upon the work of Hal Anger, who solved the problem of geometrically analyzing gamma emissions from whole organs, Ron Jaszczak and his colleagues developed the first whole body SPECT camera in 1976 (4). The first medical study of functional brain activity with SPECT neuroimaging was published in 1978 (5).

Unlike the X-ray, computed tomography (CT) and magnetic resonance imaging (MRI), SPECT did not become a standard of care in the fields of medicine devoted to the treatment of the functioning and malfunctioning brain—Psychiatry and Neurology, except for a brief period in the 1980s when brain SPECT was used to evaluate the consequences of stroke and later Alzheimer's disease. Over forty years after the development of SPECT neuroimaging, these fields of medicine still ignore a valuable tool for examining the functional status of the brain. One must ponder the question—why?

## Brain Function and Psychiatry in History

Ironically, Sigmund Freud, the father of psychoanalysis and its attendant attribution of human behavior to ephemeral intrapsychic agencies, such as the Id and Ego, actually began his inquiry into human behavior by trying to understand the functioning of the human brain (6). As detailed in his letter to Wilhelm Fliess, Dr. Freud described impacts upon the nervous system and explored the effects of sinus pathology upon the workings of the brain—and ultimately the psyche. Unfortunately, he did not have the tools to understand brain function and its direct relationship to human behavior, and he became frustrated.

The psychoanalytic movement that Freud sparked resulted in the brain being ignored by psychiatry. This was to the detriment of patients, including the notable Ukrainian-American composer, George Gershwin, who died in 1937 of a slow-growing brain tumor which was not caught earlier because his psychiatrist was completely focused on trying to uncover the psychoanalytic underpinnings of Gershwin's severe headaches. His psychiatrist did not think or act like a doctor of medicine. He ignored Gershwin's symptoms of sudden memory lapses, olfactory hallucinations of the smell of burning rubber, bouts of incoordination, and other clear signs of a neurological disturbance. Gershwin is but one of thousands who died needlessly because psychiatrists ignored the brain.

## Psychiatry in the Modern Era

But now we live in a more enlightened time. We recognize the role of the amygdala, the thalamus, the hippocampus, the dorsolateral prefrontal cortex, and the insula in neuropsychiatric function. We recognize that lesions to the frontal cortex can disrupt judgement, motivation, and social decorum. Psychiatric meetings and presentations are peppered with pictures of the brain and often a functional MRI or two. Yet, none of this neurobiological awareness has translated into the ***practice*** of psychiatry. Psychiatrists seem to rely entirely on their intuition to decide what is wrong with a patient. Some experts state psychiatrists make a diagnosis in less than 15 minutes of patient interview (7). Treatment decisions seem to be determined by the psychiatrist's clinical experience, rather than scientific evidence supporting clinical efficacy (8). If a patient appears similar to a previous patient, then the newly diagnosed patient is more likely to get the same medication that worked for the previous patient (8, 9).

Mind you, there are diagnostic criteria for the diagnoses established in Psychiatry. The Diagnostic and Statistical Manual V (DSM-V) provides a set of symptoms and signs which must be present to give a patient a certain diagnosis (10). Most of these criteria are subjective, and the overlap between diagnoses can be striking. For example, it is very difficult to diagnose a patient with a personality disorder without having sufficient diagnostic criteria to meet the DSM diagnostic criteria for, yet, a second personality disorder. Moreover, the diagnostic system of the DSMV was created by committees and is artificial. Therefore, it is not surprising that fully 60% of the DSMV diagnoses failed to stand up to validity testing when subjected to field trials (11). Dr. Thomas Insel, then-head of National Institutes of Mental Health, stated (12, 13):

The DSM-IV has 100% reliability and 0% validity … We need to develop biomarkers, including brain imaging, to develop the validity of these disorders … Trial-and-error diagnosis will move to an era where we understand the underlying biology of mental disorders … We are going to have to use neuroimaging to begin to identify the systems pathology … to develop treatments that go after the core pathology, understood by imaging.

In other words, the artificial groupings and separations of symptoms do not match the real world experience, nor do they match the neurobiological evidence. Nassir Ghaemi, MD, noted expert on psychopharmacology recently wrote (14):

“Psychiatry … practice(s) non-scientifically; we use hundreds of made-up labels for professional purposes, without really getting at the reality of what is wrong with the patient…

The DSM system which dominates Psychiatry has other problems. Multiple physiologically distinct entities or phenotypes are lumped together under a single diagnostic label. For example, using the DSM diagnostic criteria for Major Depressive Disorder (15), there are over 20 possible distinct phenotypes of this single diagnosis. Patients with depression can have insomnia or hypersomnia. They can have increased motor activity or decreased motor activity. They can have weight gain or weight loss. It is difficult to see how such a multi-faceted presentation can represent a single diagnostic entity which would benefit in the same way from the same medications and/or treatments. Indeed, many experts agree that depression is not simply one thing, despite the efforts of mainstream psychiatry to classify it into a single illness category.

The inadequacies of DSM categorical diagnostic strategies have been highlighted by the relative failures of multiple large treatment trials, including the STAR*D, STEP-BD, and the CATIE (16–19). These massive multi-million dollar clinical trials largely showed meager clinical benefit of newer psychotropic medications and a general lack of ability to make and keep patients well. As Thomas Insel stated (20),

“current medications help too few people to get better and very few people to get well.”

The STAR*D trial (16, 21) examined a sequenced protocol of antidepressant medications for patients with depression who had failed at least one prior medication trial in an open-label protocol with no placebo control. In this large multi-center trial of over 2,876 patients, all of the patients were initially treated with citalopram. Non-responders were then switched to a stepped sequence of other medications. At step 2, patients were prescribed either buproprion, sertraline, venlafaxine or buproprion or buspirone augmentation of citalopram. At step 3, patients were prescribed mirtazapine, nortriptyline, lithium augmentation or triiodothryonine augmentation. In step 4, non-responders were randomized to receive tranylcypromine or a combination of venlafaxine and mirtazapine. In addition to an extraordinarily low final remission rate of 13% (22), STAR*D demonstrated decreasing response rates, increasing rates of intolerable side effects and increasing likelihood of relapse (23). For example, of the 1,085 patients who had a positive response to citalopram in Step 1, 92% experienced relapse within 12 months despite continued pharmacotherapy.

As such, the STAR*D trial demonstrated: (1) the failure of a DSM psychiatric diagnosis to predict an effective treatment and (2) the overall failure of standard antidepressants to yield lasting clinical benefit. Moreover, the STAR*D results forced study researchers to acknowledge, “that major depressive disorder is biologically heterogeneous, such that different treatments differ in the likelihood of achieving remission in different patients” (21).

By contrast, neuroimaging studies have revealed several neurophysiological substrates for depression. Functional brain scans, such as SPECT (single photon emission computed tomography) or PET (positron emission tomography) have shown that patients presenting with the same symptoms of depression can have very different functional features occurring in their brains (24). Indeed, some of the anatomic circuits of depression and mood regulation have been revealed by converging evidence from SPECT, PET, and fMRI studies of depression, as well as the analysis of the effects of either traumatic lesions resulting in depressive symptoms or surgical interventions used to treat severe cases of depression (25, 26). A network of brain regions have been revealed by convergent neuroimaging findings, which includes the dorsal prefrontal cortex, ventral prefrontal cortex, anterior cingulate gyrus, amygdala, hippocampus, striatum, and thalamus, and together contribute to the pathophysiology of depression (27–29). Experts have emphasized that depression is the result of multiple pathophysiological processes and the dysfunction of multiple pathways (25, 30). Depression is not a singular disorder and is unlikely to have a singular treatment.

The distinct subtypes of depression detected using functional neuroimaging do indeed predict and demonstrate distinct treatment responses. In a large proportion of depression cases, decreased activity (and therefore metabolism and perfusion) of the frontal lobes, the insular cortex, and the anterior cingulate gyrus (25–30) is found. However, some patients with depression have increased perfusion in the precuneus, which correlates with rumination and self-criticism (31). In contrast, some patients with depression also have decreased temporal lobe function. Many patients with depression show increased thalamic activity (metabolism or perfusion) (32). Portions of the thalamus have direct connections to the amygdala, the seat of fear, and anxiety (33). Functional neuroimaging, such as SPECT and PET, can also predict who will respond to certain antidepressants. For example, those who are likely to respond to SSRI antidepressants show increased perfusion in the ventral frontal cortex and anterior cingulate (34, 35). SSRI antidepressants often induce decreased activity and perfusion in these areas, as well as in the thalamus. In contrast, some patients with depression have markedly decreased dorsal frontal cortex and medial frontal cortex activity and perfusion. These patients are less likely to respond to SSRI medications, but may respond better to noradrenergic antidepressants (29, 36). Treatment-resistant depression may show markedly increased activity and perfusion in the subgenual cingulate (30).

## Neuroimaging Could Guide the Diagnostic Process—If Only…

Ironically, in this age of heightened awareness of the importance of the brain and its central role in the generation of the symptoms, we associate with psychiatric disorders, the American Psychiatric Association (APA) has taken the position that neuroimaging has no role in Psychiatry. In 2018, the APA published a position paper stating “neuroimaging has yet to have a significant impact on the diagnosis or treatment of individual patients in clinical settings” (37).

When the APA officially declared that using neuroimaging to look at the brain ***has no clinical value in psychiatry***, it took a step backwards scientifically. As if to codify the aphorism, “The Absence of evidence is evidence of absence”, the APA then conveniently ignores an entire body of neuroimaging research which will be elaborated below. It also took a giant leap backward in terms of the evolution of the medical diagnostic process. It is not an overstatement to say that an obvious step in the diagnostic process inherent to all forms of medicine is to actually look at the organ of medical concern. A surgeon would not think of operating—even under the most emergent of conditions—without first obtaining an image of the surgical region. Cardiology uses SPECT neuroimaging as a cornerstone of the diagnostic evaluation of the heart. The APA also took a step backwards morally by denying patients a potentially better way to arrive at the treatment plan. Is it truly better to guess or depend upon intuition versus utilizing all possible forms of diagnostic information at one's disposal? Are multiple failed trials of antidepressants better for the patient?

The APA's claim that neuroimaging has not had a significant impact on the diagnosis and treatment of psychiatric illnesses seems to assume that functional brain neuroimaging can only be helpful if it provides a pathognomonic “fingerprint” for a DSM diagnosis. Notwithstanding the absurdity of expecting imaging of the human brain to yield a hallmark of a disorder created by a committee, issues of comorbidity, and the shared final neurophysiological outcome of multiple “diagnoses” make it highly unlikely logically that we will have neuroimaging “fingerprints” for committee-created disorders.

Moreover, comorbidity (the presence of two or more diagnoses) is the rule, rather than the exception in psychiatry. Patients with ADHD frequently have comorbid anxiety, oppositional disorders, or learning disorders (15, 38–40). Patients with depression have a very high rate of comorbid anxiety (15, 41). Patients with Post-Traumatic Stress Disorder (PTSD), particularly veterans, often have comorbid Traumatic Brain Injury (TBI) (42–46). These comorbid diagnoses cloud the diagnostic process. The DSMV was not designed with the brain in mind and has done little to adopt the lessons learned about the neurobiology of psychiatric disorders. Furthermore, functional aspects of the brain do not neatly fit into DSMV categories (15).

Perhaps most shocking is the fact that this official declaration by the APA (37) was not open to debate. There were no hearings of interested parties. There was no opportunity for rebuttal. The leadership of the APA made a mandate. The mandate is that Psychiatry will not look at the brain of patients as part of their evaluation, diagnostic workup, and care. The mandate is to ignore the changes in the brain—both positive and negative—that could be induced by the prescribed treatments. The mandate is to ignore that other physiological processes may be occurring which masquerade as a DSM psychiatric diagnosis. The mandate is to ignore a large body of medical evidence that neuroimaging does improve psychiatric care. And in so doing, the leadership of the APA is depriving themselves, psychiatrists, and the general public of effective and promising functional neuroimaging opportunities that could improve clinical care. Of great concern is the fact that such a respected organization would take a position that misleads the profession and denies physicians and their patients a potentially useful adjunct in the process of arriving at a treatment plan. This becomes especially concerning in light of the opinions of experts in the field and the supporting research that the DSM categorical diagnostic system is clearly flawed—and we must look for a more brain-based diagnostic system.

## Missed Opportunities in Psychiatric Neuroimaging

As members of the International Society of Applied Neuroimaging (ISAN), we cannot agree with the position of the APA. Our clinical experience as psychiatrists, neuroimaging specialists, nuclear medicine physicians, and experienced general practitioners is that functional neuroimaging, using SPECT and/or PET functional imaging, provides valuable insights into patient diagnosis. We will now summarize the data that support the use of SPECT and/or PET functional neuroimaging in the diagnostic process, as well as in the treatment monitoring process. In addition, we will demonstrate how other pathophysiological processes can masquerade as psychiatric conditions. Only by correctly determining the absence of these confounding pathophysiological processes can treatment be maximally effective.

Ultimately, we will demonstrate that functional brain imaging with PET and SPECT has already proven itself valuable in the psychiatric treatment of individual patients, and we will illustrate how it can contribute to the advancement of the field, going forward.

## The Incongruity Between DSM Diagnostic Categories and Neuroimaging Findings

Besides the case of depression, another example of the incongruity between DSM diagnostic categorization and neuroimaging data is the diagnosis of Attention-Deficit-Hyperactivity Disorder (ADHD). There is overwhelming neurobiological and neuroimaging evidence that multiple forms of ADHD exist. A multitude of functional imaging studies utilizing a diversity of modalities, including SPECT, fMRI, PET, and quantitative electroencephalogram (qEEG) repeatedly derived similar results in children and in adults. Some of these studies, reviewed by Cherkasova and Hechtman (47), showed reduced regional brain activity during a concentration task. Areas such as the prefrontal cortex, orbital frontal cortex, and caudate nuclei in some patients with ADHD decrease activity during concentration. This is what we have come to recognize as “typical” or “intrinsic” ADHD.

However, there are alternate neurobiological processes which produce an ADHD-like phenotype. For example, abnormal anatomy and function have been reported in the cerebellum of some patients with ADHD. Others diagnosed with ADHD have poorly functioning temporal lobes (47). Our significant clinical experience has shown that patients with symptoms of ADHD can also present with diffuse over-activity of the cerebral cortices, involving not only the frontal lobes, but also the temporal and parietal lobes. This clinical observation is supported by recent research looking at subclasses of ADHD endophenotypes (48, 49). Neuroimaging data clearly argues there is more than one form of ADHD. Moreover, comorbidity occurs with high frequency in patients with ADHD. Those with ADHD frequently have comorbid anxiety, oppositional disorders, or learning disorders (24, 38, 39). Lastly, traumatic brain injury (TBI) involving the frontal lobes can result in impulsivity, impaired attention, reduced judgment, and other hallmark symptoms which are indistinguishable from ADHD. It would be foolish to expect an injured brain to respond in the same manner as an intact brain.

Altogether, these observations argue that we should not expect all patients with the diagnosis of ADHD to respond favorably to the same medications. As we have previously described, alternate endophenotypes of ADHD have been revealed by neuroimaging, and the medication responses of these individuals is different. For example, we have described patients who became agitated and aggressive on stimulant medications (24). SPECT functional neuroimaging revealed widespread over-activity throughout the cerebral cortices—a finding inconsistent with intrinsic ADHD. These patients respond favorably to anti-convulsant medications with a reduction in hyperactivity, impulsivity, agitation, aggressiveness, and inattention.

A second example of an alternate endophenotype in ADHD is reduced temporal lobe function (47). These patients will often demonstrate no evidence of frontal lobe deactivation during a concentration task, but persistent or worsening temporal lobe hypoperfusion with SPECT functional neuroimaging. Given that acetylcholine is an important neurotransmitter in the temporal lobes, we have utilized donepezil (Aricept), an acetylcholinesterase inhibitor for these patients. Donepezil was shown in a small study to improve ADHD symptoms (50). In patients with reduced temporal lobe function, donepezil improves attention and academic performance (24).

A third and highly prevalent example of an alternate endophenotype for presumptive ADHD is TBI involving the frontal and/or temporal lobes. Among patients with TBI, problems with attention and concentration occur in at least 50% of cases (51–54). Injury to the neurons of the frontal lobes leads to neuronal dysfunction. While this can resemble the decreased function of frontal lobe regions found in intrinsic ADHD, it is unrealistic to expect these injured neurons to respond to medication in the same manner as healthy neurons. By the same token, toxic brain injury can also masquerade as ADHD. Frontal lobe neuronal injury from a host of toxins and other insults can lead to impaired attention and other symptoms of ADHD. This will be explored in greater detail in the next section. Suffice to say that one need only look to the headlines to see evidence of the increase in diagnosis of ADHD in areas afflicted by lead toxicity (55).

## Pathophysiological Subterfuge—How Toxicity, Infection, and Inflammation Masquerade as Psychiatric Diagnoses

An important underlying concept for diagnostic imaging in all its forms is that imaging allows a clinician to eliminate possibilities and narrow the differential diagnosis. This progression is central to any medical diagnostic process. Physicians obtain laboratory values to eliminate alternative explanations for a patient's symptoms and focus in on the diagnosis. Similarly, physicians order imaging studies to further eliminate alternate diagnoses and close in on the actual diagnosis for that particular patient. For example, if a patient presents with shortness of breath which is worse with exertion, such as when climbing stairs, then a diagnostic process is begun. Let us examine that process from two perspectives and by comparing it to the symptom of anxiety. First, we will approach this patient as a psychiatrist would. Second, we will approach this patient in the manner that most physicians in medicine would.

A psychiatrist will start by interviewing the patient. Regardless of the primary symptom—shortness of breath or anxiety, the psychiatrist might ask when does this happen? What makes it worse? What makes it better? Are there any associated symptoms? Perhaps, what does this mean to the patient? Interestingly, a patient with shortness of breath may often become anxious. After listening carefully, then psychiatrist would then label the primary symptom as a diagnosis (shortness of breath disorder or anxiety disorder) as a diagnosis. The psychiatrist might pontificate upon the causes, rooted in childhood trauma or recent losses or the psychiatrist might simply prescribe a medication based upon what was discovered in the interview. At this point, the cause of the shortness of breath (or anxiety) is still unknown, and therefore, the chances of curing the ailment are quite small. Nonetheless, the patient leaves with a prescription for a benzodiazepine and may indeed have some transient relief of the symptoms of shortness of breath and anxiety. Yet, the patient is no closer to a diagnosis, an effective treatment, or a cure than they were when they walked into the psychiatrist's office.

A physician in other medical disciplines will start by interviewing the patient. Many of the same questions will be asked. But then the physician digs deeper. The physician will examine the patient, listen to the lungs, try to induce the symptom (shortness of breath or anxiety) in the office, and order laboratory tests. The physician will likely order imaging studies—a chest X-ray, perhaps a cardiac stress test (a form of SPECT scan), perhaps a V-Q scan to rule out pulmonary embolus. Ultimately, with the combination of history, physical exam findings, laboratory testing results, and imaging results, the physician will arrive at a short list of diagnostic possibilities and will ask more questions of the patient or order further tests to arrive at a cause for the original symptom. Is the patient short of breath due to pneumonia, bronchial obstruction, cardiac insufficiency, or something else? Is the patient anxious due to shortness of breath, cardiac insufficiency, paraneoplastic syndrome or a dozen other possible etiologies? The treatment would then be tailored to the etiological basis of the symptom. A cure could be forthcoming.

We can hear our psychiatric colleagues scoff. “Most people with anxiety have an anxiety disorder”, they would say. “Most people with alternating high and low moods have bipolar disorder”, they would add. Why go on a wild goose chase?

We can understand their incredulity. For decades, psychiatry has not had to consider alternative etiologies for their diagnoses. There was no challenge to the categorical DSM system; however, now, if psychiatry is to move forward, it must challenge the categorical DSM system and incorporate the findings of evidence-based neuroimaging into psychiatry. In the way of a brief example, following the advent of anatomical MRI, routine assessment of first episode psychosis included a brain MRI or CT scan. By the late 1990s this practice was largely abandoned with the justification that the expense did not justify the return on finding an organic cause (reviewed in 56). Unfortunately, alternative etiologies such as toxicity or infection, likely did not show up in anatomical MRI and were, therefore, missed.

Today, there is substantial evidence for the role of toxins and infections, such as *Toxoplasma gondii* or viral infection, in schizophrenia. There is growing evidence of immunological dysfunction causing psychosis (57, 58). The changes in brain function associated with these infections can show up on functional SPECT scan. Newer PET tracers for brain inflammation are now being explored. Thus, looking at the brain with functional neuroimaging in cases of psychosis may strongly suggest a ***treatable cause*** for the psychotic symptoms. The functional brain scan may lead the physician to laboratory studies, which definitively reveal an infection or inflammatory process. As a result, a patient could be treated with appropriate antibiotics or anti-inflammatories targeting the cause of the disorder. Rather, than condemning a patient to a lifetime of antipsychotic medications, which may or may not help, a more biological approach might cure the patient.

Schizophrenia is not the only example of a disorder with possible immunological or infectious causes. Significant evidence supports the role of infections and inflammation in obsessive-compulsive disorder, anxiety disorders, depressive disorders, and possibly bipolar disorder. Recognizing the DSM diagnoses are clusters of symptoms and not actual biological entities is essential to being able to look for treatable causes of brain dysfunction, which currently are lumped together into singular DSM diagnoses. Neuroimaging can and does play a critical role in this process. As Thomas Insel, stated:

Imagine deciding that EKGs were not useful because many patients with chest pain did not have EKG changes. That is what we have been doing for decades when we reject a biomarker because it does not detect a DSM category. We need to begin collecting the genetic, imaging, physiologic, and cognitive data to see how all the data—not just the symptoms—cluster and how these clusters relate to treatment response (13).

We will explore some of the biological and, therefore, treatable contributors to psychopathology which can and are visualized using functional neuroimaging.

## Auto-Immune

It has long been recognized that auto-immune disorders, such as systemic lupus erythematosus can cause neurological and psychiatric symptoms. Over the past two decades, appreciation has grown for a widening array of auto-immune disorders with very specific psychiatric manifestations. For example, autologous antibodies to epitopes upon the neurons of the basal ganglia can be produced following a Streptococcal infection. The resulting damage to the function of the basal ganglia can lead to severe anxiety and obsessive-compulsive symptoms (59, 60). This syndrome, referred to as Pediatric Autoimmune Neuropsychiatric Disorders Associated with Streptococcal infections (PANDAS) has now been found not only in children, but in adolescents and adults (61). In addition, it has been found to occur following other non-strep infections (62, 63). The more generalized term for this condition is Pediatric Acute-onset Neuropsychiatric Syndrome (PANS). The symptoms include: abrupt onset of anxiety or obsessive-compulsive symptoms, emotional lability or depression, irritability, aggression, motor and sensory abnormalities, urinary dysfunction, academic decline, and sleep disturbance. A patient with one or more of these symptoms could be variably diagnosed with obsessive-compulsive disorder, depression, bipolar disorder, oppositional defiant disorder, intermittent explosive disorder, anger dysregulation disorder of childhood, and/or generalized anxiety disorder (10). However, a functional brain SPECT scan may reveal diffuse hypoperfusion throughout the cerebral cortices and increased activity in the basal ganglia. This pattern would not be expected for any of the psychiatric diagnoses above. Rather, this finding signals a diffuse insult to the brain, such as a toxic, inflammatory, or infectious attack.

## Infections

A growing body of evidence suggests that infections with cytomegalovirus (Herpes 5), Epstein-Barr virus (Herpes 4) or Human Herpes virus 6 (Herpes 6) are responsible for a portion of cases of depression and/or bipolar disorder (64–67). Prusty and colleagues (65) examined samples of human cerebellum from a brain bank for Herpes 6 DNA. Using immunofluorescent labeling, followed by fluorescent in-situ hybridization (FISH), as well as DNA amplification by polymerase chain reaction (PCR), the group localized Herpes 6A and 6B virus in the tissue. Cell type was confirmed by co-staining with immunofluorescent markers specific to cerebellar Purkinje neurons and for glial cells. The striking finding was that Herpes 6 DNA and signs of DNA replication were found within neurons from human brains. The distribution of Herpes 6-positive neurons was not uniform. Among brains from patients with the diagnosis of depression (N = 25), 53% were positive for Herpes 6B DNA, while only 24% of those with bipolar disorder (N – 25) and 16% of control brains (N = 50) contained DNA of the Herpes 6B virus. Prusty and colleagues (65) have provided definitive evidence that Herpes 6 virus can reach the brain in a significant proportion of humans and replicate therein. The group's findings also provide a strong association between the presence of Herpes 6 DNA and mood disorders. As yet, causality has not been demonstrated.

Clinical data do support, but also do not prove, a causal relationship between Herpes viruses and mood disorders. For example, Henderson (67) found high levels of antibodies against Herpes 4 and Herpes 6 in adolescents diagnosed with treatment-resistant depression. Anti-viral therapy with valacyclovir resulted in marked improvement of fatigue and depressive mood symptoms. Recently, Frye and colleagues (66) reported increased levels of Herpes 5 antibodies in patients diagnosed with bipolar disorder. SPECT functional brain scans of patients affected by these types of infections reveal diffuse hypoperfusion, a marker of infection, toxic injury, or sometimes inflammation. The scans of such patients typically do not reveal increased thalamic activity, a marker often associated with depression.

## Inflammation

Furthermore, the role of inflammation in depression is now supported by a wealth of evidence. Experts now believe that inflammatory processes underlie and contribute to depressive pathophysiology in a significant proportion of cases (68–71). These concepts grew from observations that medications which reduced inflammatory processes had antidepressant properties. For example, treating multiple sclerosis with powerful anti-inflammatories was associated with reduction of depressive symptoms (68). Conversely, treatment of hepatitis C with interferon alpha, a powerful activator of an inflammatory response, induces depression in roughly 20% of patients (72, 73). Work over the last decade has characterized the nature of the inflammatory processes underlying depression and its possible influence on the brain. Briefly, cytokines are elevated in a portion of patients suffering from depression. In particular, tumor necrosis factor alpha (TNFα), interleukin-2 (IL-2), interleukin-6 (IL-6), interleukin-13 (IL-13), interleukin-18 (IL-18), C-reactive protein (CRP), chemokine-2 (CCL2), and chemokine-11 (CCL11) are elevated in depression based on multiple meta-analyses (68, 71, 73). But how inflammatory cytokines induce depressive symptoms remain incompletely understood.

There is evidence that TNFα alters endothelial cells which make up the blood-brain barrier leading to increased permeability. Thus, cytokines have access to the CNS and can induce CNS inflammation. Elevated levels of TNFα and other cytokines have been detected in the hippocampus and striatum and can induce impairment of long-term potentiation in the hippocampus leading to depressive symptoms (74). Cytokines also appear to suppress the response to reward cues in the ventral striatum (75). Efforts to reduce inflammation using anti-cytokine medications have shown promise as anti-depressants. Kappelmann and colleagues (76) recently summarized the state of the field in a meta-analysis. For example, TNFα antagonists had anti-depressant effects in a subset of patients who had elevated CRP levels (69, 70, 76). The IL-6 antagonist, tocilizumab, also shows anti-depressant qualities (76). Similarly, rituximab, an antibody which inhibits B cell activity, can induce decreased fatigue and improved mood in patients treated for rheumatoid arthritis (73). Recently, the antibiotic, minocycline, which has immunomodulatory effects, has been shown to improve depressive symptoms (77). Altogether, several meta-analyses now support the role of inflammation in a portion of depression cases. Treating the inflammation could potentially eliminate or “cure” the depressive symptoms. However, how would the psychiatrist ever know to treat for inflammation? Inflammation is unlikely to be revealed by an interview question.

The inflammation that underlies depression in these cases often leads to diffuse hypoperfusion on functional SPECT brain scan. More striking are cases of marked increased perfusion in early inflammation which presents a paradoxical finding not encountered in cases of depression. Recently developed, but as yet not commercially available PET markers for inflammation will show marked increased uptake in these cases (70, 78). As described above, this is distinct from the pattern of increased thalamic activity which often characterizes depression. Treating a patient so affected with a serotonin reuptake inhibitor or other antidepressant is unlikely to provide optimal response. In contrast, treating the inflammation may eliminate the depressive symptoms. However, for the psychiatrist to know which approach to utilize, he/she would benefit from first looking at the brain function.

## Gut-Brain Interactions

While controversial, the role of the gut and gut bacteria (microbiome) is gaining appreciation as a contributor to psychiatric symptoms, including mood symptoms. The various yeasts and bacteria species that make up the microbiome live in a delicate balance. Antibiotics, excessive sugar, alcohol, environmental agents, and foreign infections can destabilize this balance. Overgrowth of certain bacteria or yeast species can damage the tight-junctions of the lining of the gastrointestinal (GI) tract leading to abnormal serum levels of polysaccharides and other molecules normally confined to the intestinal tract. Inflammatory responses within the GI tract can lead to pathological irritation of the vagus nerve with resulting depressive symptoms (79, 80). GI tract inflammation appears to also be capable of inducing CNS inflammation (79).

While as yet poorly studied, many clinicians are utilizing antifungals for depression, anxiety, schizophrenia, behavioral dysregulation, and autism (81, 82). While skeptical, the authors have experience with patients whose depression, agitation, irritability, and manic-like mood symptoms improved with elimination of sugars and a course of probiotics and antifungals. This emerging field is often missed in interview and abnormal functional brain scan patterns could lead the psychiatrist to alternative organic causes, which might include gut-brain dysfunction.

## Illustrative Case 1

We offer an illustrative example with the understanding that the plural of anecdote is not data. A male college student presented for evaluation due to problems with attention, feelings of anxiety, getting confused in social or high-stimulus situations, getting lost or feeling uncertain about his route when going to class, and academic decline. The outside psychiatrist diagnosed the young man with Bipolar Disorder, Social Anxiety Disorder, and ADHD. After several medication trials which seemed to have no benefit or make the patient feel worse, the patient withdrew from college and returned home. As the patient worked in psychotherapy with the psychiatrist and after the patient became irritable and agitated on a psychostimulant, the psychiatrist added the diagnosis of Borderline Personality Disorder.

The patient came to the clinic of one of the authors (TAH) for a second opinion. Notably, the young man had gone from being a socially active and motivated high school student to being reclusive, lethargic, unmotivated, and demoralized. Careful interview and additional symptom questionnaires revealed profound fatigue, unrefreshing sleep, and low-level confusion. The academic failure, getting lost or confused about routes, and the inattention were more consistent with “brain fog,” a colloquial term for mild cognitive dysfunction.

**Figure 1:**
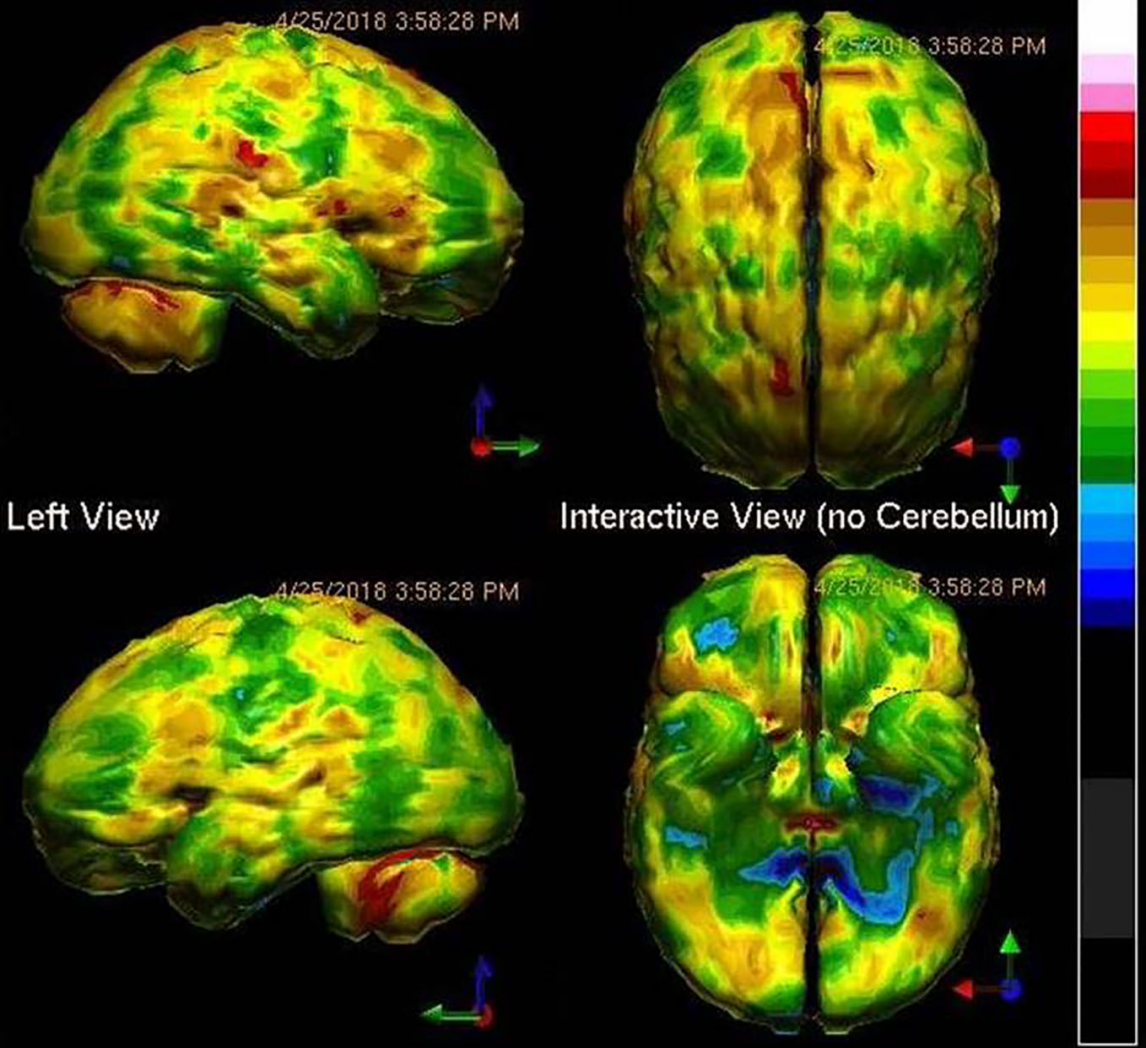
Tc-99m-HMPAO perfusion SPECT scan data presented in surface rendering. The color scale is scaled relative to the patient's mean cerebral perfusion. Mean blood flow (72%) is in yellow. Color shifts occur at approximately every 0.5 SD (3%) relative to the patient's mean. Diffuse cortical hypoperfusion (green and blue) is clearly evident.

A search for a biological cause of the patient's symptoms was clearly in order. A SPECT functional brain scan was ordered. The result is presented in **Figure 1**. The SPECT scan revealed diffuse hypoperfusion, suggesting toxic, infectious, or inflammatory processes. The patient denied substance abuse, including the huffing of chemicals. His toxicology screen was negative. Viral antibody levels were ordered, and these revealed no elevation of antibody levels for Herpes 1,2,4,5, but elevated antibodies to Herpes 6. The C-reactive protein level was normal. The patient was started on antiviral therapy (67). However, after four months there was little change in his symptoms. Perhaps the first psychiatrist was correct. The patient had bipolar disorder and borderline personality disorder.

Yet, the SPECT scan told a different story. Therefore, testing was done for Borellia, Bartonella, and Babesia infections. While, the patient was negative for Lyme's disease, he was positive for *Bartonella henselae* and *Babesia duncani*. Antibiotic therapy was started. Within two months, the patient made a remarkable turn-around. Anxiety diminished. Concentration and focus improved. His energy and motivation improved. He felt clear-headed and could now recognize how “foggy” he had been. The patient got a part-time job and resumed college classes. Rather than being condemned to a lifetime of powerful psychotropic medications with considerable potential side effects and diagnostic labels which carry considerable negative connotation, the patient underwent a course of antibiotic therapy and returned to a normal life. Without the SPECT scan, we would not have been guided to look for an infectious cause. Multiple interviews by psychiatrists had missed these infections. No DSM-guided interview question would have prompted a psychiatrist to look for these infections. Without neuroimaging, the diagnosis would have been missed, and the treatment would have been incorrect.

## The Single Most Common Biological Cause of Psychiatric Symptoms

Not surprisingly, the single most common masquerader of psychiatric disorders is TBI. Brain trauma irrefutably leads to disruption of the function of the affected portion of the brain. Since the brain is highly organized with each structure involved in a specific function, the functional disruption depends on what part of the brain is injured. Damage to the parietal lobes can affect visuospatial processing, spatial orientation, and language/writing abilities. Damage to the temporal lobes, which is among the most common injuries (83), leads to memory difficulties, learning difficulties, and emotional dysregulation. Damage to the frontal lobes, the single most common area to be injured (83), leads to impaired judgment, impaired concentration, fatigue, depression, and a host of other challenges.

The role of functional neuroimaging and brain SPECT, in particular, extends beyond showing the location and extent of local abnormality at the site of impact, which is typically seen as areas of hypoperfusion. Indeed given the brain's capability to compensate by recruiting other structures both proximal and distal to the impact site, one may be faced with areas of higher than normal perfusion, as well. Such areas may often overcompensate (over-recruit) and can present as localized areas of extreme hyperperfusion, frequently at distance from the site of impact. The importance of this is that such hyperperfused structures may generate and sometimes dominate the patient's symptoms and thus need to be considered when establishing a tailored treatment plan (84). Alternatively, TBI may present with diffuse perfusion changes on brain SPECT. This can be seen, for example, in MVA where there has not been direct contact between skull and hard objects, but only effects of significant acceleration/deceleration. In the context of clinical history this type of diffuse brain SPECT pattern can be most helpful in the process of evaluating and treating patients with TBI (84).

The link between TBI and psychiatric symptoms is well-characterized. For example, over 40% of patients who experience a concussion (also known as mild TBI) will develop depression over the subsequent year (85, 86). Other conditions may develop as well. Concussion and TBI can lead to depression, suicidal ideation, anxiety, irritability, anger outbursts, relationship problems, irrational or socially inappropriate behaviors, cognitive changes, and impaired memory. How does a psychiatrist differentiate these symptoms from the symptoms of Depression, Anxiety, Bipolar Disorder, Post-Traumatic Stress Disorder, or Dementia? There is no single interview question that will differentiate TBI from any of these diagnoses.

Questionnaires designed to assess psychiatric disorders often have tremendous overlap with symptoms of post-concussive or chronic TBI. For example, the Zung Depression Scale (87) assesses disrupted sleep, mental clarity, fatigue, irritability, decision making, and anhedonia—all symptoms found in patients with TBI. Similarly, the Zung Anxiety Scale (88) assesses nervousness, tremor, headaches, dizziness, tingling in the limbs, and disrupted sleep—all symptoms common for patients with TBI. Several of the question items within the Clinician-Administered PTSD scale (89) can be symptoms of TBI, such as sleep difficulties, poor concentration, memory difficulties, social isolation, anhedonia, and irritability.

Naturally, including questions specifically about TBI in all psychiatric interviews will increase the likelihood of identifying a patient who may have TBI masquerading as a psychiatric disorder. Caution is warranted as often patients with TBI and anterograde amnesia will not recall a head injury when asked the first time. Only with multiple inquiries into a history of head injuries, might a persistent clinician get a positive answer. At this point, the clinician might seek functional neuroimaging to clarify the presence, location, and the magnitude of abnormality and its contribution to the patient's symptoms.

The situation has particular significance for the nation's veterans. TBI and PTSD are both common in military populations, creating a particularly thorny challenge to distinguish the two conditions (46). These two conditions may overlap by as much as 33% to 42% (90). Recently, the Veterans Administration facilities revealed 73% of patients reporting TBI were comorbid for PTSD (91). For patients who have both TBI and PTSD, which generate many of the same symptoms, the VA acknowledges that the patient is often diagnosed with only one of the conditions (42–45). Indeed, the VA admitted in November 2017 that it had misdiagnosed tens of thousands of veterans (92).

Brain injury alters the way the brain responds to its environment, to stressors, and to medications. There is no reason to expect patients with TBI to respond to medications in the same way as those who have endogenous depression. The pharmacological treatments for TBI are largely targeted towards symptoms rather than the cause of neurological disruption (93, 94). These pharmaceuticals include the serotonin reuptake inhibitors, serotonin-norepinephrine reuptake inhibitors, benzodiazepines, mood stabilizers, and atypical antipsychotics (93, 94). Several studies have examined the benefit of sertraline for post-TBI depression (95–97). Other serotonin reuptake inhibitors also have been examined (94). The results are not consistent, and those with a history of TBI are more likely to be deemed treatment-resistant.

Modulation of the dopaminergic system may improve alertness, attention, and cognitive processing speed. The stimulants are most commonly used for this purpose. Amantadine and bromocriptine may also increase dopamine. Studies of these agents have shown reduced abulia, anergia, and anhedonia in those with TBI (98, 99). However, amantadine may cause confusion, hallucinations, and hypotension. Small studies have suggested some benefit of bromocriptine on cognitive function (100, 101).

While antidepressants can be useful in managing some of the symptoms of TBI, the prescribing of benzodiazepines to those with TBI can impede function or be dangerous (94, 102, 103). Of note, antipsychotics have been shown to impede recovery or be dangerous in clinical studies and animal models of TBI (104, 105). Yet, a factor associated with an increased likelihood of a veteran being prescribed an antipsychotic or a benzodiazepine is the diagnosis of TBI. For example, 41% of veterans with PTSD were prescribed benzodiazepines, while those with both PTSD and TBI had a 67% chance of being prescribed a benzodiazepine. Similarly, antipsychotics were prescribed to 25% of veterans with PTSD, but 40% of those with both PTSD and TBI (103). Other treatments for PTSD, such as transcranial magnetic stimulation, can be dangerous in TBI due to induction of seizures (106).

Hyperbaric oxygen treatment has been explored as a treatment for TBI. Hyperbaric oxygen therapy is neither a completely benign treatment, given the concerns of oxygen toxicity (107, 108), nor is it a clear treatment in that the placebo condition of moderate hyperbaric room air also effectively improves cognitive function (109). The most carefully performed study compared a group in a cross-over design with both an interval of null treatment and of hyperbaric oxygen at 100% oxygen and 1.5 atmospheres (110). They described improvement in many of the symptoms associated with persistent TBI including headache, tinnitus, vision disturbance, memory dysfunction, and impaired cognitive function. Hyperbaric oxygen remains a controversial area both in acute TBI (107–109) and for some in chronic TBI (111, 112), while others have found hyperbaric oxygen is quite helpful as a treatment for TBI (113).

Unfortunately, little has been found to reverse the damage of TBI or repetitive concussion which is the root cause of residual cognitive and psychological impairment following TBI (94). One potential avenue of treatment for TBI is infrared light, which has shown promising data in a number of applications (114, 115). Transcranial application of near-infrared light using multi-Watt infrared laser has shown efficacy in treating multiple symptoms of TBI, including depression, anxiety, sleep disruption, hyperarousal, memory problems, and sleep disruptions (42–45, 51, 116).

## Illustrative Case 2

A second case illustrates this situation well. Based on a review of the chart notes prior to the patient coming to one of the author's clinic, the patient presented in the following manner. A young adult male with the diagnosis of depression was referred to a well-seasoned psychiatrist. The referral from a psychologist was prompted by the findings on psychological testing that suggested a Bipolar-type mood disorder. Specifically, the patient was given a Minnesota Multiphasic Personality Inventory (MMPI) which showed an introverted man with low morale and depressed mood. The MMPI also indicated a preoccupation with feeling guilty and worthless, that he deserves to be punished for the wrongs in his life, that he struggles to manage daily affairs, and has poor memory, poor concentration, and no energy for life. He scored high on certain schizophrenia subscales, as well as on introversion and depression subscales. The psychiatrist conducted an evaluation interview and elicited statements consistent with depression, difficulty concentrating, and social withdrawal. A history of a trial of citalopram for depression and a low dose of risperidone for “possible Bipolar Disorder” was elicited. The psychiatrist inquired about medical problems, but did not ask specifically about TBI. After thirty minutes, the psychiatrist diagnosed the patient with Major Depressive Disorder, Social Anxiety Disorder, and Somatoform Disorder. He was prescribed citalopram. Later, he switched the patient to fluoxetine and then added mixed dextroamphetamine salts.

The patient continued on this regimen for over two years, but not really feeling much better than he had at baseline. He then went to another psychiatrist for a second opinion. In the sixty minute evaluation interview, the second psychiatrist elicited a history of a TBI, but did not pursue it further. The patient was switched from fluoxetine to buproprion and continued on the stimulant medication. Yet, the patient remained socially isolated, moderately depressed, amotivated, pessimistic, and resigned to a meager life of solitude. He worked as a landscaping assistant, but did not socialize outside of work with peers or coworkers. He avoided family functions because it was too over-stimulating. He struggled with memory issues and difficulty making decisions.

**Figure 2:**
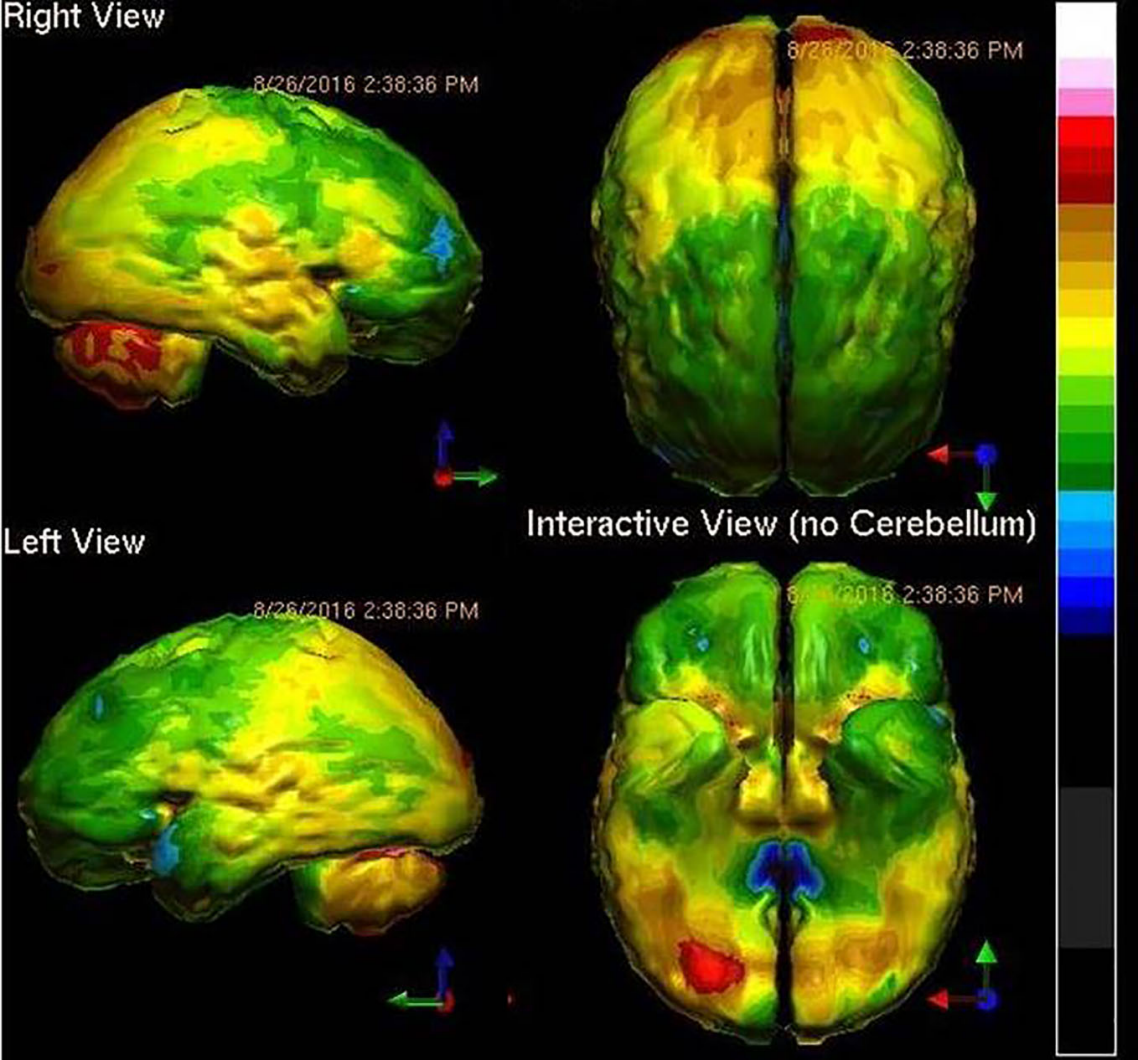
Tc-99m-HMPAO perfusion SPECT scan data presented in surface rendering. The color scale is as in **Figure 1**. Bilateral frontal and temporal hypoperfusion (green and blue) is clearly evident and is consistent with traumatic brain injury.

Three years later, he came to one of the authors for a third opinion. In this interview, the details of the TBI were explored in more detail. The patient reported he had been in a high speed collision at age 17 and had been thrown through the windshield. He reported that he had been unconscious off the road for some time before he was found. He reports that he has no recollection of the accident or the events for many weeks afterwards. Moreover, he has no recollection of his childhood or his high school years. His family has told him that he had to relearn how to use eating utensils, read, write, and talk. The history was sufficient to warrant getting neuroimaging to determine what parts of the brain were injured. **Figure 2** shows the patient's SPECT scan.

The case illustrates not only the degree to which TBI can affect a person's life, but also the unfortunate tendency among psychiatrists to underappreciate the degree to which TBI can generate psychiatric symptoms. The patient shows damage to the frontal and anterior temporal lobes, as well as to the insular cortices. Thus, the symptoms of low motivation, struggles with decisions, poor concentration, depression, anxiety, low energy, impaired memory, and introversion all make sense. Add to this neurophysiological insult, the loss of all memories prior to the accident, and the patient begins to look like an entirely different case. An SSRI and a stimulant are unlikely to fix this patient's symptoms.

## Functional Neuroimaging Can Aid the Diagnostic Process

While the position of ISAN is not that SPECT or PET functional neuroimaging replaces the diagnostician, it is our position that functional neuroimaging can aid and inform the diagnostic process and resulting treatment plan. Can neuroimaging provide a pathognomonic imaging result (a fingerprint, if you will) for each DSM condition? No, but it can eliminate several possibilities and lead one closer to a definitive conclusion. Functional neuroimaging can offer clues and information about psychiatric disorders and their comorbid conditions.

Functional neuroimaging helps clinicians to unravel complex cases. For example, ruling out toxic exposure or TBI can be highly valuable in the differential diagnosis of complex cases. The ability of SPECT neuroimaging to differentiate TBI from PTSD not only meets the APA criteria, but offers hope to tens of thousands of veterans who suffer from one or both disorders (42–45). As a second example, if a perfusion SPECT scan shows a diffuse pattern of decreased function, ADHD become much less likely and systemic effects such as metal (117), mold (118), or other (119) toxicity, carbon monoxide poisoning (120), or infection (121) become more likely. Rather than treating the patient with a stimulant, a clinician would be directed by the scan results to seek a cause for the brain dysfunction, as described above.

There are diagnoses for which SPECT and/or PET functional neuroimaging have proven their value for thorough evaluation and the guidance of effective treatment strategies. Indeed, the sensitivity and specificity of SPECT and PET meets or exceeds the APA recommendations in a number of disorders.

## Well-Established Diagnostic Roles for SPECT and PET

**First,** numerous published, peer-reviewed studies by independent investigators show that both fluorodeoxyglucose positron emission tomography (FDG-PET) and SPECT meet the criteria set by the APA in diagnosing Alzheimer's disease (122). FDG-PET and SPECT both have sensitivity and specificity in the diagnosis of Alzheimer's disease between 82% and 89% (122). Moreover, FDG-PET and SPECT are superior to amyloid imaging in the differential diagnosis of the various forms of dementia (24, 122). To be specific, a positive amyloid scan can reliably be considered evidence of Alzheimer's disease or its precursor (sensitivity = 89%; specificity = 87%) (123). However, the non-specific binding in amyloid scans increases dramatically with age to over 40% in patients over 80 years (122, 124). Thus, the specificity of amyloid imaging declines dramatically with age. Furthermore, if an amyloid scan is negative, then the patient presumably does not have Alzheimer's disease, but it is impossible to differentiate among the alternative forms of dementia with an amyloid scan. FDG-PET and SPECT are superior in this regard (24, 122) and can provide clear evidence to aid in the differential diagnosis.

**Second,** brain perfusion SPECT (125, 126) can readily distinguish ADHD from controls. Indeed, SPECT scans differentiated children who were highly likely to respond favorably to stimulants from non-responders (125). As illustrated above, SPECT neuroimaging can provide important clues which aid in the clinical management of presumptive ADHD cases, which do not respond in a typical fashion to stimulant medications. Nonetheless, Lee and colleagues (125) characterized a reasonably sized sample of 40 medication-naïve children with ADHD compared to 17 controls using SPECT plus statistical parametric analysis before and after treatment with methylphenidate. Statistical analysis confirmed that subjects with ADHD showed decreased perfusion (activity) of the prefrontal cortex and middle temporal gyrus, but showed increased perfusion (activity) in the somatosensory cortex and anterior cingulate gyri, compared to controls. After treatment with methylphenidate, ADHD subjects showed increased perfusion of the prefrontal cortex relative to their own pre-medication scans. Perfusion in the somatosensory cortex and striatum was reduced (125). These SPECT studies have been confirmed by numerous fMRI studies which have found similar impairment of the fronto-striatal networks. For example, Pliszka and colleagues (127) found that adolescents with ADHD (N = 17) failed to show increased perfusion (activation) in the anterior cingulate bilaterally and the left ventrolateral prefrontal cortex during an inhibitory task (Stop Signal Task) compared to 15 age-matched controls. Smith and colleagues (128) similarly described decreased perfusion in the left rostral mesial frontal cortex during one interference-type concentration task and decreased perfusion in the bilateral inferior prefrontal (right more significant than left) and temporal lobes during a switch task (128).

**Third,** perfusion SPECT brain function neuroimaging is very useful in differentiating TBI from controls and TBI from PTSD (42–45, 129, 130). Notably, there is tremendous overlap (33% to 42%) (90, 91) between the clinical presentation of TBI and PTSD in veterans. As discussed above, diagnostic instruments routinely used by the Veterans Administration (VA) are neither sensitive, nor specific. For instance, several of the symptoms assessed by questions in the Clinician-Administered PTSD scale (89) could be a result of TBI, such as sleep difficulties, irritability, poor concentration, and memory difficulties. Using perfusion SPECT neuroimaging, TBI and PTSD can be differentiated with a sensitivity of 92% and a specificity of 85% based on a study of 196 veterans (129). Furthermore, these results were replicated in a separate civilian population of over 24,000 individuals (130). These findings certainly meet the APA's criteria for a psychiatric “biomarker,” which they declared should have greater than 80% sensitivity and specificity and be replicated with independent data.

**Fourth,** independent groups have shown that SPECT neuroimaging (or its equivalent) improves treatment outcomes. In a six-month open-label outcome study of 500 patients, SPECT scans were associated with improved clinical outcome (23). Of the 500 patients, 231 were diagnosed at baseline with depression and had overall tried 5.45 different medications and seen four prior psychiatrists. After six months of multi-modal treatment (pharmacology, nutrition, lifestyle change) guided by SPECT scan results, over 56% of the depressed patients had a greater than 50% improvement in depression levels as assessed by the Beck Depression Inventory (23). Quality of life measures also were markedly improved (85%) in patients whose treatment was guided by functional neuroimaging findings (23).

Similar results were obtained in a smaller study by a different group (36). A group of 28 patients who underwent SPECT functional neuroimaging at baseline and received treatment guided by the results of the SPECT scans were compared to a matched group of 28 controls who received treatment guided only by clinical acumen. The information derived from the SPECT scan, in combination with clinical information, determined the course of pharmacological treatment or changes to the pre-existing pharmacological treatment. After a course of treatment, patients underwent a repeat SPECT scan. Duration of treatment was variable in this open-label, naturalistic study (mean = 17 months). The primary outcome measure was GAF, and no additional quantitative assessments were routinely included. Nonetheless, improvements in GAF were greater for those patients whose treatment was guided by SPECT scan results (13.8 vs. 8.6; p < 0.01).

Cerebral perfusion can also be measured using arterial spin labeling in an MRI and was recently shown to predict SSRI response (131). In a sample of 231 patients with Major Depressive Disorder, increased perfusion in the putamen, anterior insula, inferior temporal gyrus, parahippocampal gyrus, inferior parietal lobule, and the orbitofrontal cortex predicted a positive response to the SSRI antidepressant sertraline (131). Further studies of the value of cerebral perfusion neuroimaging, using SPECT and arterial spin labeling in guiding effective treatment are ongoing.

## Limitations of SPECT and PET Imaging

We certainly do not want to whitewash functional neuroimaging and acknowledge that there are some limitations and areas of potential concern. If neuroimaging is accepted and adopted by the psychiatric community, then several of the current limitations will resolve as part of the evolution of the use of neuroimaging as a modality in the field. These malleable current limitations include: 1) reluctance of insurance companies to cover the procedure, 2) unrealistic expectations of a pathognomonic “fingerprint” for a DSM diagnosis on the part of patients and physicians, and 3) the lack of comfort, understanding, and familiarity among physicians with the relationship between neuroimaging findings and neuropsychiatric conditions as elaborated in this review. We foresee this progression mirroring the process by which other neuroimaging modalities, the DaTscan for Parkinson's disease and the amyloid scan for Alzheimer's disease, have gained acceptance by the insurance industry. As neuroimaging finds its place as a tool in the evaluation and ongoing assessment of neuropsychiatric patients, greater cooperation between nuclear medicine physicians and psychiatry physicians will evolve naturally. Further replications of the work by Thornton and colleagues (36) and others to definitively demonstrate that neuroimaging improves treatment outcomes would likely attract the attention of third-party payers. In fact, SPECT neuroimaging arguably meets the criteria set forth by the Centers for Medicare & Medicaid Services for Coverage with Evidence Development classification and providing Medicare coverage for psychiatric indications. SPECT is already covered by Medicare for TBI and dementia.

Education of physicians will be essential in this process of introducing neuroimaging into mainstream psychiatry. This is no different from the situation of introducing the first atypical antipsychotic, the first dopamine partial agonist, or transcranial magnetic stimulation. The expectations and the understanding of both patients and physicians will come through education. Thematic to this review has been highlighting the disparity between the categorical system of diagnosis and the neurobiological findings.

In our collective clinical experience, patients tend to be less interested in the “diagnosis,” compared to physicians. Rather, patients tend to be most interested in learning what can be done for their condition, whatever it might be. SPECT and PET functional neuroimaging helps the patient by revealing the functional substrate of their psychiatric/psychological symptoms. There are two very fascinating outcomes we have observed. First, the patient, as well as their family, engages in less blaming and criticism since they now see the symptoms as the product of disrupted neurophysiology. Second, the patient often demonstrates improved compliance with treatment.

Since both SPECT and PET neuroimaging are nuclear medicine techniques, which involve radioactive tracers, there is an associated risk of radiation exposure. By contrast, MRI and functional MRI do not expose the patient to radiation. Unfortunately, these non-invasive MRI modalities have failed to yield clinically useful diagnostic approaches (15, 37). A brain FDG PET scan on average carries a 700 mRems exposure (132), while a head CT scan can lead to an exposure of 800 to 900 mRems (132), depending on the imaging protocol. A perfusion SPECT scan of the brain carries a 640 mRems exposure (132). This exposure is approximately two times the range of typical background exposure (290–390 mRems) from the natural environment and modern technology, such as smoke detectors, televisions, computers, and air travel (133). The radiation exposure from a SPECT scan is less than that of cardiac fluoroscopy for stent placement, abdominal fluoroscopy, or helical cardiac CT imaging (134). A pediatric SPECT scan uses considerably less tracer and yields an average exposure of 220 mRems (134). This is less than a CT scan, abdominal fluoroscopy, or other pediatric fluoroscopic procedures (134).

Many physicians lack a frame of reference for these numbers and concerns around any radiation exposure permeate medical thinking. However, the fear that any amount of radiation exposure can lead to adverse outcomes is not actually supported by the medical literature (134). Without delving too deeply into the theoretical debate, the question boils down to whether or not there is a Linear No-Threshold Model or a Threshold Model for risk associated with radiation exposure. The proponents of the Linear No-Threshold Model hold that all radiation is potentially harmful, as summarized by Howe and McLaughlin (135) and the BEIR V report (136). However, on close examination, the studies often cited as supporting the Linear No-Threshold Model, in fact, do not support the Linear Model. In particular, Howe and McLaughlin (135) reports increased cancer rate among women exposed to chest fluoroscopy, but these data clearly demonstrate a decreased risk at doses below 2000 mRems (135, 137). Likewise, a study by the International Agency for Research on Cancer (138) involving 95,673 subjects demonstrated a *negative correlation* between low-level radiation exposure and cancer risk. Indeed, the cancer risk was increased only at radiation doses exceeding 4,000 mRems (138).

Additionally, there is extensive evidence against long-term risk associated with low dose radiation exposure (139–141). For example, Saenger and colleagues (139) reported on a three year follow-up of 18,379 patients treated with radioactive iodine for hyperthyroidism with an average dose of 10,000 mRem to bone marrow, relative to non-irradiated subjects, and found no increased rate of leukemia. Long-term risk does not appear to be elevated in children either. For example, Ron and colleagues (140) described a cohort of 11,000 patients under the age of 15 years treated with radiation for tinea capitis and receiving an average dose of 9,300 mRems (range, 4,500–50,000 mRems). Compared to 16,000 controls at 22-year follow-up, there was no difference in the rate of leukemia. In summary, the risk of cancer or radiation injury associated with SPECT scans with a radiation dose of only 608 mRems or PET scans at a radiation dose of 700 mRems remains hypothetical, as there is no published data demonstrating an actual quantitative increase in cancer rates associated with nuclear brain scans.

But what do the experts say about the overall risk of undergoing a SPECT scan or a PET scan? A leading authority on the subject of medical radiation exposure, Dr. Michael Devous, has stated in several settings (142, 143),

“…that there are no data that have ever demonstrated any harm to humans by radiation exposures at **diagnostic imaging levels** (emphasis added). In fact, current data support the presence of radiation hormesis; that low levels of radiation exposure induce beneficial effects of cellular repair and immune system enhancement….Therefore, it should be concluded that neither SPECT nor PET brain imaging procedures are associated with any particular risk over activities of daily living and certainly should not be considered to be any more risky than MRI or any of its associated functional imaging derivatives”.

Along the same lines, the Health Physics Society in their 2004 and 2009 position papers (144) states,

“the Health Physics Society recommends against quantitative estimation of health risks below an individual dose of 5,000 mRems in one year or a lifetime dose of 10,000 mRems … There is substantial and convincing scientific evidence for health risks at high-dose exposure. However, below 5,000 to 10,000 mRems (which includes occupational and environmental exposures) risks of health effects are too small to be observed or are non-existent”.

Similarly, the American Nuclear Society in its 2001 position paper (145) states,

“There is insufficient scientific evidence to support the use of the Linear No Threshold Hypothesis (LNTH) in the projection of effects of low-level radiation.”

## Conclusion

One could assume that the introductory sentence of the APA position paper clearly signals the fear that drives the APA's position (37):

“In response to claims being made that brain imaging technology had already reached the point at which it could be useful for making a clinical diagnosis and for helping in treatment selection in individual patients”

If we reframe this “driving fear” as trepidation about abandoning a categorical system of diagnosis and undertaking the process of learning about and of transitioning to a clinically useful diagnostic process that includes functional neuroimaging, then the resistance of the APA becomes more understandable. The present psychiatric clinical disorder system, DSMV, was never designed to enable the psychiatrist to envision the underlying neurobiological function of the disorders that they are “seeing” clinically. The challenge of pulling away from a categorical system and transitioning to one that takes infections, inflammation, brain injury, toxic injury, and possibly even microbiome influences, into account might be chilling to the average psychiatrist, as well as to the leadership of the APA. But, as Thomas Insel (13) quoted Craddock and Owen (146) stated,

“at the beginning of the 21^st^ century, we must set our sights higher”.

Functional neuroimaging is and must continue to be at the center of efforts to unravel the neurobiology of psychiatric illness and provide help for its treatment.

Those of us who use neuroimaging in our daily clinical care of patients, we have come to appreciate what is only recently being voiced by the research community, namely that functional neuroimaging reveals biotypes within psychiatric illness that both subdivide and cross DSM categorical boundaries. In his introduction to the July 2019 issue of the APA's lead journal which was devoted (ironically) to neuroimaging in the wake of the APA edict, Kalin (147) called upon Psychiatry to develop brain-based, data-driven protocols for diagnosis and treatment targeting the structurally and functionally distinct neural targets to:

“significantly improve the lives of many of our patients who continue to suffer”.

In the same issue, Etkin (148) described the “missed opportunities” of neuroimaging in Psychiatry as including not recognizing earlier the absence of a neural signature for categorical DSM diagnoses. He encouraged functional neuroimaging studies with larger N's and a perspective of seeking indicators of treatment response, similar to his work with cerebral perfusion described above. He also advocates for data sharing across centers and the increased use of the single-patient, multiple-repeated-measures study design to elucidate neural mechanisms underlying key symptoms and key treatment responses. Etkin stated: (148)

“we may be at an important inflection point for the field”.

Insel had recognized and advocated for this inflection point almost two decades earlier stating: (149)

Patterns of regional brain activity associated with normal and pathological mental experience can be visualized … and ultimately, biomarkers for mental disorders may not be proteins or neurotransmitters but may emerge from neuroimaging (functional magnetic resonance imaging (fMRI), single photon emission computed tomography (SPECT), etc. Logically, if these are disorders of brain systems, then the visualization of abnormal patterns of brain activity should detect the pathology of these illnesses.

The members of ISAN renew their call upon the APA and psychiatrists everywhere to re-examine functional brain imaging in Psychiatry with inclusion of SPECT and FDG-PET research and their already published ***clinical utility***. Rather than set unrealistic APA expectations for a neuroimaging biomarker for a psychiatric disorder and its comorbid conditions, we encourage the APA to appreciate the already-recognized value of using functional brain imaging in the incremental steps of a clinical differential diagnosis and to elucidate biotypes underlying key symptoms rather than seeking DSM categorical fingerprints in neuroimaging. If Psychiatry is to take:

“an honest reflection on trends and approaches the field has taken to date, and reckoning with their assumptions and impact”

as Etkin (148) asked of the field, we may come to agree with him that we are at “an important inflection point for the field.” That inflection point is the acceptance of functional neuroimaging as a diagnostic tool, not to stand alone, but to aid, direct, and guide the ordering diagnostician to a better and more efficient evaluation and treatment of the neurobiological processes that underlie a particular patient's symptoms.

## Author Contributions

TH was involved in organizing, drafting, writing, and editing the manuscript. ML was involved in organizing, writing, and editing the manuscript. MM was involved in organizing, writing and editing the manuscript. JU was involved in image processing and figure preparation, as well as organizing, writing, and editing the manuscript. JT was involved in organizing, drafting, writing, and editing the manuscript. Y-HS was involved in organizing, writing, and editing the manuscript. DP was involved in organizing, drafting, and editing the manuscript. JC was involved in organizing, drafting, and editing the manuscript. PC was involved in organizing, drafting, writing, and editing the manuscript.

## Conflict of Interest

MU is the Medical Director of DrSpectScan.org, a clinical service corporation and derives 25% of his income from neuroimaging. DP is Director of PathFinder Brain SPECT which is a clinical service corporation providing SPECT functional neuroimaging and derives 10% of his income from neuroimaging. Y-HS derives 10% of his income from neuroimaging. TH is the president and principal owner of The Synaptic Space, a neuroimaging consulting firm. He is also CEO and Chairman of the Board of Neuro-Luminance Corporation, a medical service company. He is also president and principal owner of Dr. Theodore Henderson, Inc, a medical service company. TH has no ownership in, and receives no remuneration from, any neuroimaging company. No more than 5% of his income is derived from neuroimaging.

The remaining authors declare that the research was conducted in the absence of any commercial or financial relationships that could be construed as a potential conflict of interest.
